# miRNA-6715-5p Inhibits Cellular Proliferation and Invasion in Colorectal Cancer by Directly Targeting CST4

**DOI:** 10.1155/2021/7615712

**Published:** 2021-05-29

**Authors:** Ding Shi, Zheng Zhou, Shun Zhang

**Affiliations:** ^1^Department of Gastroenterology, Hwamei Hospital, University of Chinese Academy of Sciences, Ningbo 315010, China; ^2^Key Laboratory of Diagnosis and Treatment of Digestive System Tumors of Zhejiang Province, Ningbo 315010, China

## Abstract

**Background:**

Data on the correlation between CST4 and colorectal cancer (CRC) metastasis are scarce. The aim of this study was to analyze CST4 expression and investigate its biological roles and related microRNA- (miRNA-) mediated regulation in CRC.

**Methods:**

The expression of CST4 was examined in cancer tissues and their corresponding adjacent normal tissues from 40 gastric adenocarcinoma patients. The expression level of CST4 in specimens (cancer and normal tissues) was assessed through immunohistochemistry and/or quantitative polymerase chain reaction. miRNAs targeting CST4 in CRC were predicted by bioinformatics software. CST4 was knocked down in HCT116 cells and candidate miRNAs were transfected into HCT116 cells, and the effects of CST4 knockdown and miRNA transfection on cell proliferation and invasion were examined using CCK8, cell colony formation, and Transwell migration assays. Luciferase double-reporter assays were performed to verify the relationship between miRNA and CST4.

**Results:**

The expression of CST4 in CRC tissues was significantly higher than that in normal paracancerous tissues, but the results for miRNA-6715-5p were opposite. Regardless of CST4 knockdown or miRNA-6715-5p overexpression, the proliferation and invasion ability of HCT116 cells decreased significantly. Luciferase double-reporter assays showed that the upregulation of miR-6715-5p significantly reduced the luciferase activities of the CST4 3′-UTR plasmid in HCT116 cells.

**Conclusion:**

CST4 may be involved in CRC proliferation and metastasis. miRNA-6715-5p directly targets CST4 and negatively regulates its expression.

## 1. Introduction

Colorectal cancer (CRC) is the most common cancer-related cause of death worldwide, mainly owing to its high rate of metastasis [1]. Because the occurrence of CRC is a complex process involving multiple genes and steps, the mechanism of its occurrence and development has not been clarified yet. Numerous studies have reported that most type 2 cystatins are involved in tumor invasion and metastasis [2–6]. In the cystatin superfamily, seven genes (CST1–5, CSTP1 and 2) have been identified. As one family member, cystatin 4 (CST4) is also known as salivary acidic protein 1 or cystatin-SA-III and regulates cysteine protease activity by specifically combining with cysteine proteases, in addition to preventing hydrolysis of the extracellular matrix [7, 8]. Previous studies have shown that not only is CST4 markedly upregulated in gastric cancer tissues, but its overexpression also significantly promotes the proliferation, migration, and invasion of gastric cancer cells [9]. The overexpression of CST4 in gastrointestinal cancer tissues and cell lines was validated as exhibiting high specificity and sensitivity, indicating that it might be a novel blood biomarker for gastric and colorectal cancer [10]. This information suggested that CST4 might contribute to carcinogenesis and tumor progression. However, the role of CST4 in CRC has rarely been reported, and thus, this role is still unclear. Further, it is also unknown if CST4 is related to metastasis in this disease. To answer these questions, we used GEPIA software (Gene Expression Profiling Interactive Analysis) to analyze the relationship between CST4 and CRC through The Cancer Genome Atlas (TCGA) database and found that its expression in CRC tissue was significantly higher than that in normal tissues (*P* < 0.05). This information inspired us to investigate the effect of CST4 on CRC metastasis.

In the present study, we investigated the expression of CST4 in CRC tissues compared with that in matched normal tissues and further elucidated its role in promoting CRC cell growth and metastasis through lentivirus-mediated CST4 downregulation experiments. At the same time, we also used bioinformatics software to predict the targeting relationship between miRNAs and CST4. Finally, we determined which miRNAs target CST4 by double luciferase assays.

## 2. Materials and Methods

### 2.1. Patients and Tissue Samples

All tissue samples were collected from the Department of Gastroenterology of Hwamei Hospital, University of Chinese Academy of Sciences, between January 2017 and October 2018. The study was approved by the Ethics Committee of Hwamei Hospital, University of Chinese Academy of Sciences. All patients were informed regarding the procedure and provided written informed consent. Each patient's surgical specimen was divided into two parts. One part was formalin fixed and paraffin embedded and then used to identify its histopathology. The other part was freshly refrigerated to be used for CST4 detection. None of the patients received chemotherapy and radiotherapy prior to tissue harvesting.

### 2.2. Cell Lines and Cell Culture

**Figure 1:**
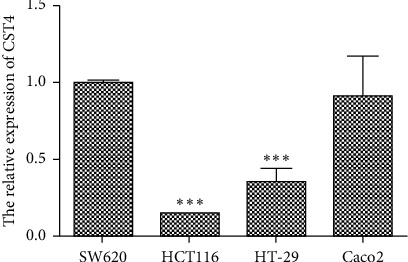
Detection of CST4 in colorectal cancer (CRC) cell lines by qPCR. The results showed that CST4 was expressed at the lowest level in HCT116 cells.

In this study, we chose HCT116 cells with the lowest expression of CST4 for HCT116 plasmid transfection. The HCT116 cell lines were purchased from the Shanghai Cell Bank (Shanghai, China). HCT116 cells with the lowest expression of CST4 were chosen for plasmid transfection (Figure 1). The cells were cultured under the following conditions: Dulbecco's modified Eagle's medium (DMEM) containing 10% FBS and 1 × penicillin-streptomycin and 37°C, 5% CO_2_, and saturated humidity incubator.

### 2.3. Transfection

The transfected plasmids included CST4 knockdown, miRNA mimic, and locked miRNA plasmids. The vector used for expressing CST4 was the pmirGlo plasmid from Life Technologies (Thermo Fisher Scientific, Inc.). For transfection, 5 *μ*L (20 *μ*mol/L) siRNA (or NC) was mixed with 100 *μ*L Opti-MEM dilution medium and let stand for 5 min. Next, the transfection reagent was mixed with 100 *µ*L Opti-MEM dilution medium and incubated for 5 min. The mixture of siRNA (or NC) and Opti-MEM was slowly dripped into the mixture of transfection reagent and Opti-MEM and was blended. The mixture was kept at room temperature for 15 min. The 200 *µ*L transfection complex was added to the well-containing cells and was gently mixed and incubated in a 37°C incubator containing 5% CO_2_ for 48 h.

### 2.4. Immunohistochemistry (IHC)

Tumor tissues were fixed in 4% formalin and embedded in paraffin. After slicing (to a thickness of 4 *µ*m) and baking (sections were placed in a 65°C thermostat for 6–12 h), IHC was performed. Sections were xylene-dewaxed and gradient alcohol-rehydrated, and endogenous peroxidase was blocked and inactivated with 3% H_2_O_2_. Next, sections were boiled for 10 min to retrieve antigens in citrate buffer (pH 6.0), cooled naturally to room temperature, washed three times with phosphate-buffered saline (PBS), and incubated with primary antibody at 4°C overnight. After rinsing three times with PBS, sections were incubated with the second antibody at 37°C for 30 min. After washing again, the sections were stained with 4′, 6-diamidino-2-phenylindole (DAPI) and then restained with hematoxylin. The staining intensity was observed and photographed, and the positive cell score was analyzed by light microscopy. Scoring criteria were as follows: 0, negative; 1–3, weakly positive; 4–5, moderately positive; 6–7, strongly positive.

### 2.5. Hematoxylin-Eosin Staining

After xylene-dewaxing and gradient alcohol-rehydrating, sections were immersed in hematoxylin dyeing solution for 5 min at room temperature. After washing in tap water for 1 min, sections were immersed in 1% hydrochloric acid alcohol solution for several seconds, and the tap water returned to blue. Next, the sections were immersed in the eosin dye solution and dyed for 3–5 min, and the float on the slide was washed with tap water. After dehydrating for 0.5 min with 80% ethanol, 95% ethanol I, 95% ethanol II, absolute ethanol I, and absolute ethanol II each, the sections were then treated with xylene I and II for 3 min each to make them clear and transparent. After removal, the sections were sealed with neutral gum.

### 2.6. Western Blot Protocol

The total cell protein was extracted according to the following steps. Samples from each group were collected into EP tubes, and 200 mL western and IP pyrolysates were added into each tube (PMSF was added before using, and the final concentration was 1 mM). After mixing, the samples were fully lysed at 4°C for 30 min and centrifuged at 4°C and 12 000 rpm, and the supernatants were collected and stored separately. Polyacrylamide gels were used for electrophoresis. First, 10% separating and 4% concentrating gels were prepared. Thereafter, the sample was mixed with 5 ×  sample buffer and placed at 100°C for 10 min and cooled rapidly in an ice bath. The sample size was approximately 30 *μ*g per lane. The electrophoretic buffer was added to the electrophoretic tank, and an 80 V power supply was connected. Constant-voltage electrophoresis was performed until bromophenol blue ran out of the glass pane. Next, 120 V constant voltage electrophoresis was used for the separating gel. When bromophenol blue migrated to the lower edge of the separation gel, the power supply was turned off and electrophoresis was stopped. After being pretreated, a PVDF membrane was inserted into the electrophoresis cell, which was transferred at a constant current of 200 mA and immersed in a sealed liquid containing 5% skimmed milk powder and sealed at room temperature for 1 h. The closed PVDF membrane was incubated with the primary antibody overnight at 4°C (the dilution ratio of CST4 was 1 : 1000 and that of GAPDH was 1 : 5000). PVDF membranes were washed three times with 1 × TBST buffer, and the secondary antibody was added and incubated for 1 h at room temperature. The blot was washed three times again with 1 × TBST buffer. Next, the PVDF membrane was placed on film, to which a mixture of a moderate amount of A solution and B solution from the ECL kit was added, and the blot was moved into the gel imaging analyzer (SMA4000, Merinton, US). The analysis software was SMA4000 V4.2.3. The chemical photosensitive mode was used, and the blot was exposed and developed. Photographs were exported in TIFF format.

### 2.7. qPCR Protocol

Total RNA was prepared using the High Purity Total RNA Rapid Extraction Kit (No., GK3016; Generay Corporation, Shanghai, China). The reverse transcription kit (HiScript- II Q RT SuperMix) for qPCR was provided by the Vazyme company (No., R222-01; Vazyme company, Nanjing). Primers for real-time PCR were designed according to the sequences of CST4 and b-actin as follows: forward primer of CST4: 5′-CCAAGGAGGAGAATAGGATAA-3′; reverse primer of CST4: 5′- AGTGAAGGGCACGCTGT-3′; amplification product of CST4: 81 bp; forward primer of actin: 5′-TGACGTGGACATCCGCAAAG-3′; reverse primer of actin: 5′- CTGGAAGGTGGACAGCGAGG-3′; amplification product actin: 205 bp. After reverse transcription, quantitative PCR was performed according to the manufacturer's instructions (ChamQ SYBR Color qPCR Master Mix, Vazyme, NanJing, Q411-02). The reaction system was as follows: 10 *μ*L SYRB Green mix, 1 *μ*L upstream primer, 1 *μ*L downstream primer, 8 *μ*L diluted cDNA, with an overall volume of 20 *μ*L. The system was mixed and then reacted with a CFX Connect Real-Time PCR (BIO-RAD) instrument. The amplification conditions were as follows: predenaturation at 95°C for 30 s, denaturation at 95°C for 10 s, annealing at 60°C for 30 s, and elongation at 70°C for 5 s, totaling 45 cycles. Actin was taken as the internal parameter, and the relative expression level of CST4 was expressed by the 2−ΔΔCt value.

### 2.8. Construction of CST4 siRNA

Three different siRNAs against the human CST4 gene were designed to specifically knockdown CST4 expression in HCT116 cells. The siRNA sequences were as follows: siRNA-1 : TATTCTCCTCCTTGGAGCTCG, siRNA-2 : ATTCACCAGGGACATTCTGTC, and siRNA-3 : TACGTCGAAGAAGTAATTCAC. The control siRNA sequence was a random small fragment with the same length.

### 2.9. Luciferase Double-Reporter Experiment

The double fluorescein luminescence was detected according to the instructions of the Dual Luciferase Reporter Assay System kit (Promega, US, E1910). The steps were as follows: (1) the 24-well plate was taken out and the culture medium was discarded; then, it was gently cleaned with 1 × PBS, and the PBS was exhausted. (2) To each well, 100 *μ*L 1 × PLB cracking fluid was added, and the plate was oscillated at room temperature for 20 min. The cracking solution was transferred to a 1.5 mL EP tube and centrifuged to obtain the supernatant. (3) Next, 20 *μ*L cracking solution was transferred to a 1.5 mL EP tube, and 100 *μ*L luciferase substrate LAR II was added into each well in the dark, and the plate was placed into the instrument for the first luciferase reading. (4) Then, 100 *μ*L Stop and Glo Reagent was added into the EP tube, which was placed into the instrument for the second reading. (5) For data analysis, the final fluorescence value of each well was the first firefly luminescence reading/the second Renilla luminescence reading.

### 2.10. CCK-8 Cell Proliferation Assay

A CCK-8 (Beyotime Institute of Biotechnology, Shanghai, China) assay was used to evaluate the growth of the HCT116 cells according to the manufacturer's protocol. HCT116 cells (4 × 10^5^ cells/well) were seeded into 96-well plates and cultured for 24 h. Next, cells were incubated with different concentrations of prucalopride (0, 0.1, 1, 10, 20, 50, 100 *μ*M) and cultured for an additional 72 h. Next, 10 *μ*L of CCK-8 solution was added into the wells, and the cells were incubated at 37°C for 1.5 h for CCK-8 detection. A microplate reader (Bio-Rad) was used to determine the absorbance (optical density, OD) of cells at 450 nm. The following experimental drug concentration (10 *μ*M) was chosen from the series of the gradient concentration. The cells were incubated with prucalopride (10 *μ*M) and vehicle (DMSO, 0.1% in culture media) for 1, 2, 3, 4, and 5 d. A CCK-8 assay was used to measure cell viability, and OD values were measured as described previously herein.

### 2.11. Colony Formation Test

HCT116 cells (200 cells/well) were seeded in a six-well plate and cultured at 37°C with 5% CO_2_ for 2 weeks. Next, the supernatant was discarded, and the cells were washed twice with PBS. Next, 0.01% crystal violet (Sigma, St. Louis, MO, USA) was added to stain the cells. After incubation for 1 h, the cells were washed three times with PBS. Finally, the cells were photographed (Olympus, Tokyo, Japan).

### 2.12. Cell Migration

After 48 h of treatment, cells were digested with 0.25% trypsin +0.02% EDTA and centrifuged, suspended with 2% serum-containing medium, counted, and allowed to migrate in 24-well plates at a 2.0 × 10^5^/well density. They were incubated with 10% serum-containing medium in the basement and 5% CO_2_ in a 37°C incubator.

### 2.13. Cell Invasion

The matrix gel was removed from the refrigerator at −20°C and was placed in an ice bath overnight at 4°C. In an ice bath, the matrix at 10 mg/mL was gently mixed with a serum-free medium of an equal volume and then added to the upper layer of a Transwell chamber and incubated at 37°C for 4–6 h. The serum-free medium was gently washed once in a Transwell chamber for later use. Cells in each group were digested with 0.25% trypsin +0.02% EDTA, centrifuged at 1500 rpm for 5 min, counted on a counter plate, suspended with 2% serum-containing medium, diluted to 5.0 × 10^5^/well, and laid in the upper chamber of a Transwell chamber at 2.0 × 10^5^/well, and cultured with 10% serum in 500 *μ*L in the lower chamber. After 16 h of culture, these cells were washed with 1 × PBS three times, fixed at room temperature with 4% paraformaldehyde for 15 min, and then washed three times with 1 × PBS again. The upper chamber cells were wiped with cotton swabs, stained with crystal violet staining for 15 min, washed three times with 1 × PBS, air-dried at room temperature, and photographed under a microscope.

### 2.14. Predicting the Targeted miRNAs Associated with CST4

miRNAs associated with CRC were identified in the NCBI (National Center for Biotechnology Information) database, and miRNAs targeting CST4 were predicted with bioinformatics software. Using CST4 as the target gene, we analyzed and compared several bioinformatics software (https://cm.jefferson.edu/rna22/Interactive/、http://www.targetscan.org/vert 71/, http://34.236.212.39/microrna/microrna/getGeneForm.do, http://mirtar.mbc.nctu.edu.tw/human/, http://129.206.7.150/) results and found nine candidate miRNAs that had the highest correlation with CST4 (multiple software predicted that this site had binding and a higher free energy score). qPCR and luciferase double-reporter experiments were carried out to validate the correlation between CST4 and nine candidate miRNAs.

## 3. Statistical Analysis

The signal intensity of protein bands obtained using western blotting or qPCR was calculated and analyzed using Quantity software (CFX Connect Real-Time System, Bio-Rad). Continuous variables are expressed as mean ± standard deviation and were compared using Student's *t*-test for two independent samples. Categorical data are expressed using absolute frequency and percentage and were compared using either Chi-square or Fisher's test. Multigroup average was calculated using CFX Manager 3.1 (Bio-Rad). The data were first tested for homogeneity of variance. If the variance was homogeneous, the differences between all groups were compared using a one-way analysis of variance. A one-to-one comparison was performed to compare the mean between the multidose group and the control group. The data of nonnormal and heterogeneous variance were analyzed using the rank sum test. Statistical analyses were performed using SPSS for Windows (version 22.0. SPSS Inc., Chicago, IL, USA). *P* < 0.05 was considered to indicate statistically significant results.

## 4. Results

### 4.1. Patient Characteristics

In total, 40 specimens were obtained from CRC and colorectal membranes near the cancer site. For 30 patients, adenocarcinoma was confirmed from surgical samples by histopathology, and regions near the cancer tissue were confirmed as normal gastric mucus membranes by hematoxylin and eosin staining. The patient age range was 41–85 years (median age of 58.7) with 16 males and 14 females. The patients' demographic data are shown in Table 1.

### 4.2. CST4 Expression Levels in CRC and Paracancerous Tissue

**Figure 2:**
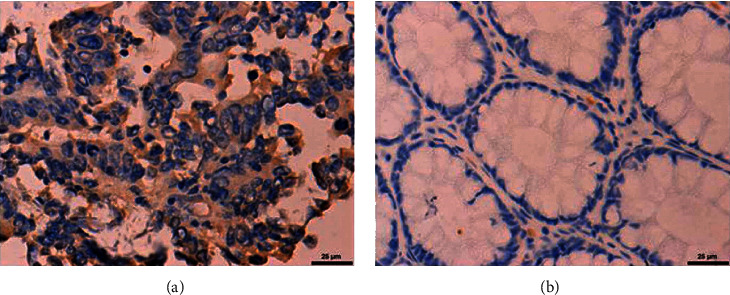
Expression of CST4 in colorectal cancer as examined by immunohistochemistry using an anti-CST4 antibody. CST4 positivity was brown or yellow in color by immunohistochemistry. Immunohistochemistry showed that CST4 staining in cancer tissue was stronger than that in paracancerous tissue. (a) Cancerous tissues; (b) paracancerous tissue.

**Figure 3:**
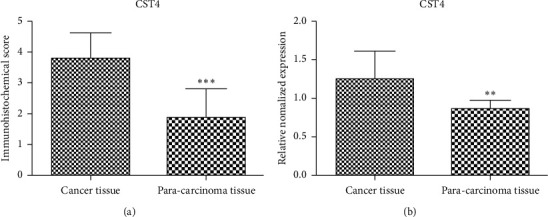
Immunohistochemistry (IHC) and qPCR detection of CST4 expression in colorectal cancer (CRC) and adjacent tissues. (a) IHC; (b) qPCR. Compared with that in adjacent tissues, CST4 expression in CRC tissues was significantly upregulated (^*∗∗∗*^*P* < 0.001, ^*∗∗*^*P* < 0.01).

IHC and qPCR were performed for 40 cases of CRC and corresponding normal adjacent tissues. CST4 positivity was indicated by a brown or yellow color by IHC. IHC staining showed that CST4 staining in cancer tissue was stronger than that in paracancerous tissue. By either IHC (*P* < 0.001) or qPCR (*P* < 0.01), the results indicated that the expression of CST4 in CRC tissues was significantly higher than that in normal paracancerous tissues (Figures 2 and 3).

### 4.3. Expression of CST4 after Knockdown in Transfected CRC Cells

**Figure 4:**
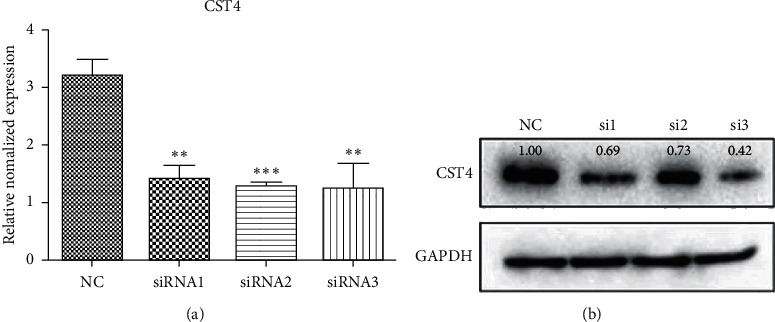
Detection of CST4 expression after a knockdown by qPCR and western blotting (WB). Compared with that in the negative control (NC), the expression of CST4 in human HCT116 cells transfected with siRNA (1, 2, and 3) was significantly decreased (^*∗∗∗*^*P* < 0.001, ^*∗∗*^*P* < 0.01). (a) qPCR; (b) WB. NC, irrelevant sequence; si1, siRNA1; si2, siRNA2; si3, siRNA3. The molecular weight of CST4 in cells was 16 kDa; the molecular weight of GAPDH cells was 42 kDa.

After knocking down CST4, compared with that in the NC, the expression of CST4 in human HCT116 cells transfected with siRNA (1, 2, and 3) significantly decreased. The results were confirmed by qPCR and western blotting (*P* < 0.001, *P* < 0.01; Figure 4).

### 4.4. Effect of CST4 Knockdown on Cell Proliferation and Invasion

#### 4.4.1. Cell Viability

**Figure 5:**
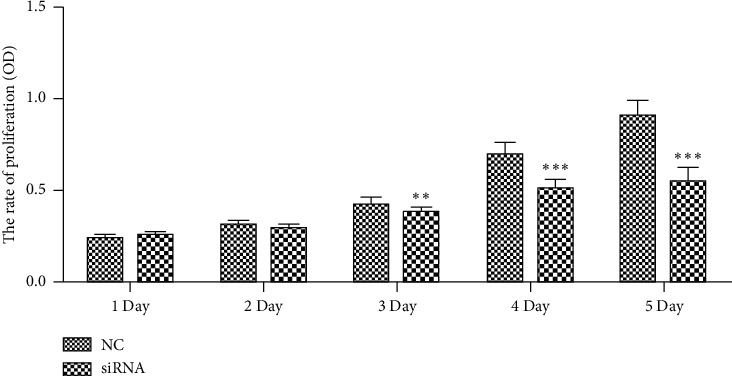
Cell viability after transfection of siCST4 at different time points based on CCK8 assays. Compared with that in the negative control (NC) at corresponding time points, CST4 siRNA-transfected cells showed a significant decrease in cell activity from the third day, and the degree of decrease increased with time (^*∗∗*^*P* < 0.01, ^*∗∗∗*^*P* < 0.001). NC, siRNA irrelevant sequence; siRNA, CST4 siRNA.

CST4 siRNA was transfected into HCT116 cells, and CCK8 was detected on 1, 2, 3, 4, and 5 d. Compared with that in the NC at the corresponding time points, CST4 siRNA-transfected cells showed a significant decrease in cell activity from the third day (*P* < 0.01), and the degree of decrease increased with time (*P* < 0.001; Figure 5).

#### 4.4.2. Cell Cloning Ability

**Figure 6:**
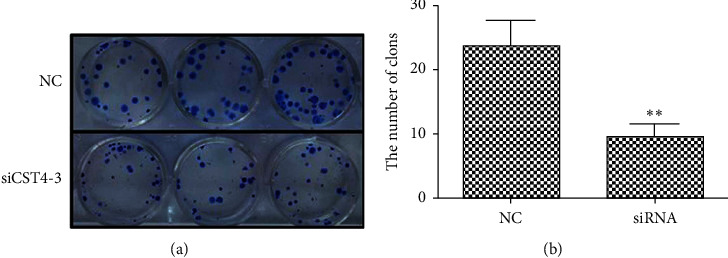
Colony formation assay results with CST4 knockdown and statistics. Compared with that in the negative control (NC), cell colony formation ability was significantly decreased after CST4 interference (^*∗∗*^*P* < 0.01). (a) Plate clone results; (b) statistics. NC, siRNA irrelevant sequence; siRNA, siCST4.

CST4 siRNA was transfected into HCT116 cells, and the unrelated sequence of the transfected siRNA was used as control. After 48 h of transfection, colony formation assays were performed. These showed that the colony growth of siRNA-treated cells was slower, fewer colonies were formed, and clones were also smaller; meanwhile, the control cells were more abundant, and colonies were larger (Figure 6). Compared with that in the NC, cloning formation was significantly decreased after CST4 interference (*P* < 0.01).

#### 4.4.3. Cell Migration and Invasion

**Figure 7:**
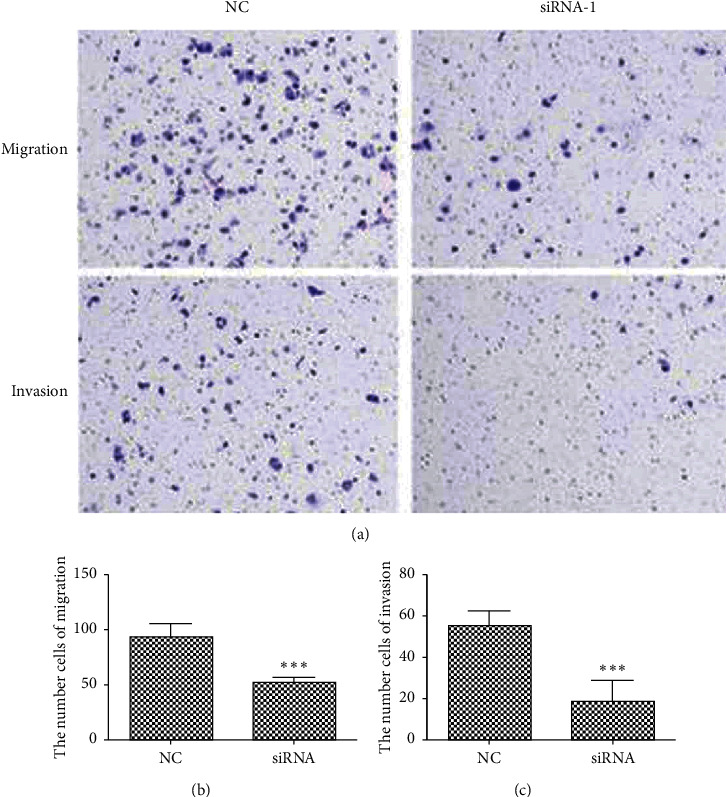
Migration and invasiveness of cells after knocking down CST4 as assessed by Transwell assays. Compared with that in the negative control (NC), the migration and invasion ability of cells decreased significantly after siRNA transfection (^*∗∗∗*^*P* < 0.001). (a) Migration and invasion results; (b) migration data; (c) invasion data. NC, siRNA irrelevant sequence; siRNA, siCST4.

CST4 siRNA was transfected into HCT116 cells, and the Transwell migration and invasion experiments were performed 48 h after transfection. The migration and invasiveness of the cells decreased significantly in the siCST4-1-, siCST4-2-, and si CST4-3-transfected cells compared with that in siNC cells (*P* < 0.001; Figure 7).

### 4.5. Expression of CST4 in HCT116 Cells after Transfecting with miRNA-Mimic

**Figure 8:**
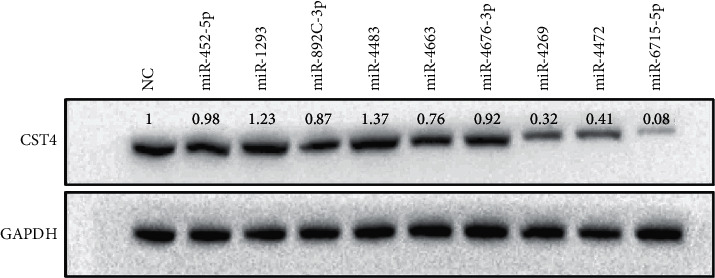
Expression of CST4 after transfection with candidate miRNAs based on western blotting. Of nine candidate miRNAs, miRNA-6715-5p caused the most significant decrease in CST4 expression. The molecular weight of CST4 and GAPDH was 16 and 42 kDa, respectively. NC, miRNA irrelevant sequence.

**Figure 9:**
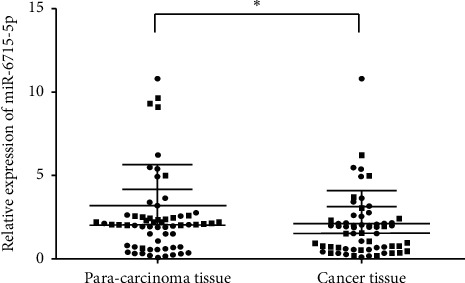
Expression of CST4 after transfecting candidate miRNAs based on qPCR. Compared with that in the negative control (NC), of the nine candidate genes, both miRNA-4269 and miRNA-4472 caused the significant downregulation of CST4 expression (^*∗*^*P* < 0.05), whereas miRNA-6715-5p caused the most significant downregulation of CST4 expression (^*∗∗∗*^*P* < 0.001). NC, miRNA irrelevant sequence.

**Figure 10:**
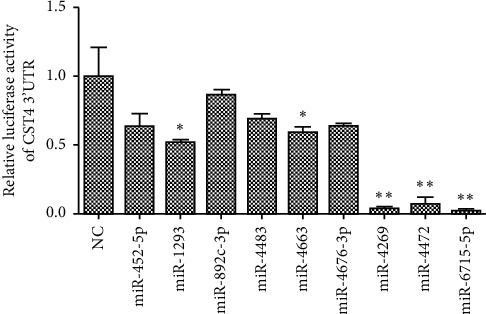
Luciferase double-reporter experiment to validate the expression of CST4 after transfecting candidate miRNAs. Compared with that in the negative control (NC), luminescence activity from the CST4 promoter in the groups treated with miRNA-4269 and miRNA-4472 decreased significantly (^*∗*^*P* < 0.05), and the most obvious decrease was with miRNA-6715-5p (^*∗∗∗*^*P* < 0.001). NC, miRNA irrelevant sequence.

Nine candidate miRNAs were transfected into HCT116 cells to detect the expression of CST4. Western blotting (Figure 8), qPCR (Figure 9), and luciferase double-reporter experiment (Figure 10) results showed that the expression of CST4 was significantly downregulated after the transfection of miRNA-6715-5p, miRNA-4269 (*P* < 0.05), and miRNA-4472 (*P* < 0.05), and especially after the transfection of miRNA-6715-5p (*P* < 0.001).

### 4.6. miRNA-6715-5p Inhibits CST4 Expression by Interacting with the Binding Site in Its 3′-UTR

**Figure 11:**
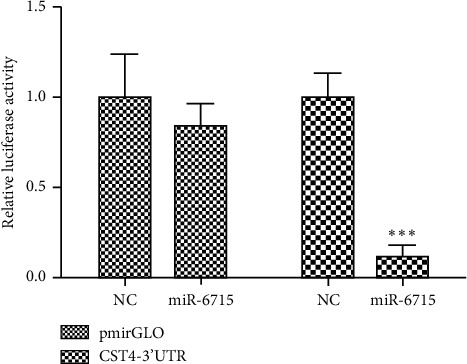
Promoter activity analysis after cotransfection of CST4 and miRNA-6715-5p. Compared with that in the negative control (NC), the luminescence activity from the CST4 3′-UTR decreased significantly (^*∗∗∗*^*P* < 0.001) after cotransfection of CST4 and miRNA-6715-5p. NC, negative control; miR-6715, miR-6715-5p mimic; pmirGlo: pmirGlo no-load plasmid; CST4-3′-UTR, pmirGlo-CST4-3′-UTR.

Luciferase double-report assays showed that the upregulation of miR-6715-5p significantly reduced the luciferase activities of the CST4 3′-UTR plasmid in HCT116 cells (*P* < 0.001). However, the luciferase activity of the pmirGlo no-load plasmid was not affected, suggesting that miR-6715-5p could directly interact with the 3′-UTR of CST4 and inhibit the CST4 gene (Figure 11).

### 4.7. miRNA-6715-5p Expression Levels in CRC and Paracancerous Tissue

**Figure 12:**
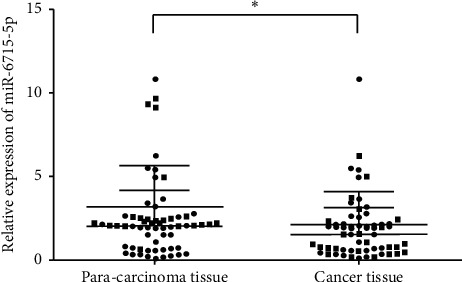
Detection of miRNA-6715-5p in colorectal cancer (CRC) and adjacent tissues by qPCR. Compared with that in adjacent tissues, miRNA-6715-5p expression in CRC tissues was significantly downregulated (^*∗*^*P* < 0.05).

qPCR was performed on 40 cases of CRC and corresponding normal adjacent tissues. The results indicated that the expression of miRNA-6715-5p in CRC tissues was significantly lower than that in normal paracancerous tissues (*P* < 0.05; Figure 12).

### 4.8. Effect of miRNA-6715-5p Transfection on Cell Proliferation and Invasion

#### 4.8.1. Cell Viability

**Figure 13:**
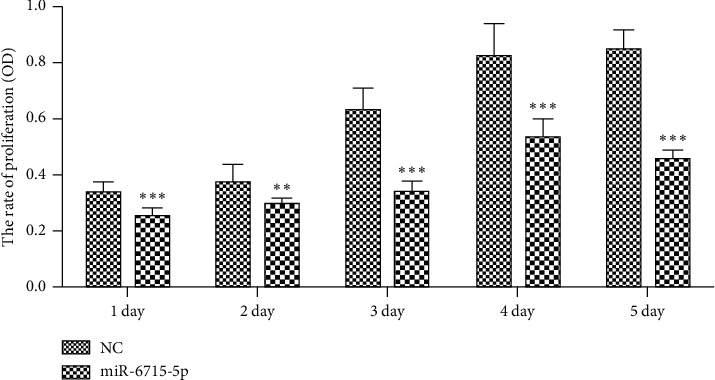
Cell viability at different time points after microRNA-6715-5p transfection based on CCK8 assays. Compared with that in the negative control (NC) at the corresponding time points, cell viability decreased significantly from the third day after miRNA-6715-5p transfection, and the degree of reduction increased with time (^*∗∗*^*P* < 0.01, ^*∗∗∗*^*P* < 0.001). NC, miRNA irrelevant sequence.

miRNA-6715-5p was transfected into HCT116 cells, and CCK8 was detected on 1, 2, 3, 4, and 5 d. Compared with that in the NC at the corresponding time points, miRNA-6715-5p-transfected cells showed a significant decrease in cell activity from the third day, and the degree of decrease increased with time (*P* < 0.01, *P* < 0.001; Figure 13).

#### 4.8.2. Cell Cloning Ability

**Figure 14:**
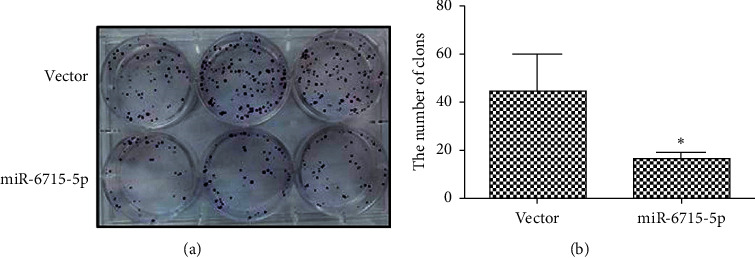
Colony formation after microRNA-6715-5p transfection. Compared with that of the cells with the empty plasmid, the colony formation ability of the cells transfected with miRNA-6715-5p was significantly reduced (^*∗*^*P* < 0.01). (a) Plate clone results. (b) statistics. Vector, empty plasmid.

miRNA-6715-5p was transfected into HCT116 cells, and the unrelated sequence of the empty plasmid was used as a control. After 48 h of transfection, colony formation assays were performed. These showed that colony growth of cells transfected with miRNA-6715-5p was slower, fewer colonies were formed, and clones were also smaller; meanwhile, the control cells produced more colonies of a larger size (Figure 14). Compared with that in the NC, colony formation was significantly decreased after miRNA-6715-5p transfection (*P* < 0.01).

#### 4.8.3. Cell Migration and Invasion

**Figure 15:**
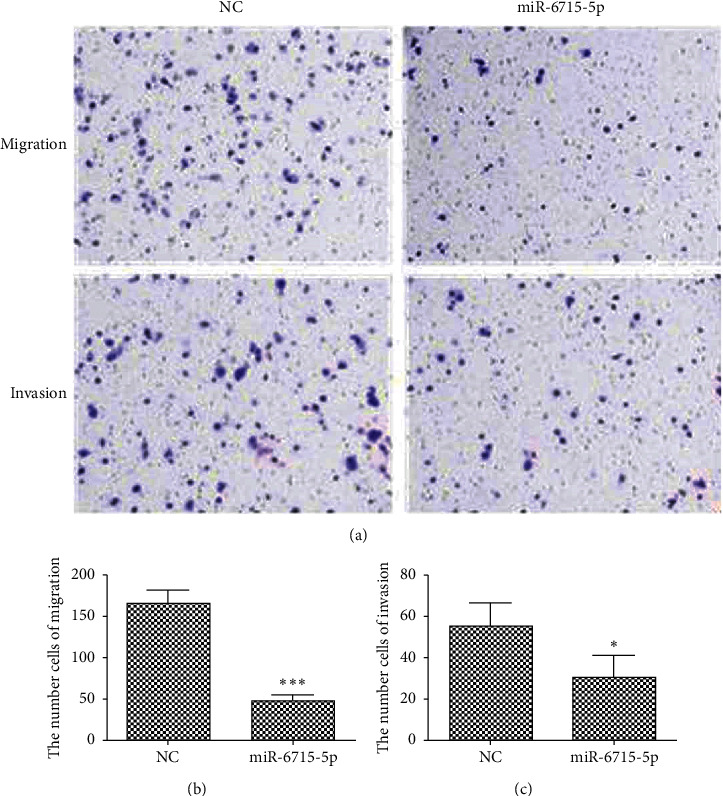
Migration and invasiveness of cells after transfection with miRNA-6715-5p based on Transwell assays. Compared with that of the negative control (NC), the migration and invasion ability of cells decreased significantly after transfecting miRNA-6715-5p (*n* = 3, ^*∗*^*P* < 0.05; ^*∗∗∗*^*P* < 0.001). (a) Migration and invasion results. (b) Migration data. (c) Invasion data. NC, empty plasmid.

miRNA-6715-5p was transfected into HCT116 cells, and Transwell migration and invasion experiments were performed 48 h later. Compared with that in the NC, the migration and invasion ability of cells transfected with microRNA-6715-5p was decreased significantly (*P* < 0.001; Figure 15).

## 5. Discussion

CRC is one of the most invasive forms of cancer and is the leading cause of cancer death worldwide. Advanced CRC is associated with significant mortality because it metastasizes to vital organs [11]. Recent studies suggest that cysteine proteases are frequently overexpressed in a variety of malignancies and contribute to cancer growth and progression [12]. In particular, type 2 cystatins are involved in the metastasis of several types of malignant tumors [13–15]. The overexpression of CST1 promotes the migration and invasion of breast cancer cells [15]. Further, CST6 is an inhibitor of osteolytic metastasis in breast cancer [16]. Moreover, high expression of CST1 and CST2 can promote bone metastasis in vivo [14]. However, data regarding CST4 are unavailable. In this study, we first analyzed the expression of CST4 in 40 CRC tissues and 40 corresponding adjacent tissues by IHC. The results showed that CST4 was significantly overexpressed in CRC tissues compared with that in normal adjacent tissues. Based on this finding, we further explored the role of CST4 in CRC growth and metastasis. As expected, the knockdown of CST4 inhibited CRC cell activity and colony formation in vivo. We also investigated the effect of CST4 knockdown on CRC cell metastasis. CST4 knockdown significantly inhibited the migration and invasion abilities of the CRC cell lines. These results suggest CST4 expression is closely related to CRC metastasis and might be a molecular target to interfere with the invasion and metastasis of CRC.

It has been reported that some miRNAs can affect the occurrence and development of CRC by regulating the expression of tumor-related target genes. miRNA-mediated gene expression regulation involves many biological backgrounds, such as cell differentiation, proliferation, apoptosis, and carcinogenesis [17–21] and can inhibit or promote the growth of CRC cells by specifically recognizing the complementary sites on the target mRNA, resulting in the negative or positive regulation of the expression of the target gene [22–26]. The low expression of many miRNA clusters in CRC regulates proliferation and metastasis by targeting specific mRNAs [27]. For example, miR-125 inhibits the proliferation and invasion of CRC by targeting PDZ-binding motif proteins [28]. However, to date, there is no report on the relationship between miRNA-6715-5p and CRC. To further study the upstream regulatory mechanism of the oncogene CST4, we used bioinformatics software to find nine candidate miRNAs with the highest correlation with this gene, and luciferase double-reporter experiments were carried out by combining these nine miRNAs with the 3′-UTR of CST4. Here, three miRNAs were found to regulate the expression of CST4 by targeting its 3′-UTR region, among which miRNA-6715-5p was most closely related to its expression.

Accordingly, we next detected miRNA-6715-5p in 40 CRC tissues and their adjacent tissues by qPCR. The results showed that its expression in CRC tissues was significantly lower than that in adjacent tissues. Therefore, it was speculated that the low expression of miRNA-6715-5p in CRC tissues is related to the high expression of CST4. To verify this hypothesis, we transfected miRNA-6715-5P into HCT116 cells and detected the expression of CST4. The results showed that the expression of CST4 in HCT116 cells was significantly decreased after transfection with miRNA-6715-5P, suggesting that the low expression of miRNA-6715-5P might be an important factor mediating the high expression of CST4 in CRC cells. At the same time, transfection of miRNA-6715-5P and knockdown of CST4 showed the same inhibitory effect on the activity, clone formation capacity, and metastasis and invasion ability of CRC cells, suggesting that there might be a negative regulatory relationship between miRNA-6715-5P and CST4. These results from luciferase double-reporter experiments further confirmed that miRNA-6715-5P has a strong inhibitory effect on CST4 expression. Accordingly, the expression of miRNA-6715-5p was negatively correlated with the expression of CST4, and the effect of both on CRC was opposite. Combined with luciferase assay results, it was suggested that miRNA-6715-5p could inhibit the metastasis of CRC by suppressing the expression of CST4.

The limitation of this study was that only in vitro experiments revealed that the upregulation of CST4 promotes the metastasis of CRC, and no evidence was provided from animal experiments. This will be investigated in our next study. In conclusion, this study provided evidence that CST4 might be involved in CRC metastasis. miRNA-6715-5p directly targets CST4 and negatively regulates its expression. Modulating the miRNA-6715-5p/CST4 axis is a potential strategy for the treatment of patients with CRC.

## Figures and Tables

**Table 1:** Clinicopathologic features of patients.

Features	Number
*Sex*	
Male	32
Female	8

*Age*	
<60	8
60–80	30
>80	2

*Histological classification*	
Adenocarcinoma	40
Lymph node metastasis	24

*Differentiated degree*	
Well	0
Moderately	24
Poorly	16

*Tumor stage*	
I	8
II	8
III	24
IV	0

*Location*	
Rectum	21
Sigmoid colon	16
Descending colon	1
Ascending colon	2

## Data Availability

The data used to support the findings of this study are included within the article.

## Supplementary Materials

Supplementary Materials Supplementary Figure 1: expression of CST4 in TGAC database. The expression of CST4 in CRC tissue was significantly higher than that in normal tissues (*P* < 0.05).
