# Supplementary material for: miRNA-6715-5p Inhibits Cellular Proliferation and Invasion in Colorectal Cancer by Directly Targeting CST4
Source: J Oncol. 2021 May 29;2021:7615712. doi: 10.1155/2021/7615712 (PMC8181091; doi:10.1155/2021/7615712)
Supplement: Supplementary Materials — Supplementary Figure 1: expression of CST4 in TGAC database. The expression of CST4 in CRC tissue was significantly higher than that in normal tissues (P < 0.05). [file 7615712.f1.docx]

**Supplementary Materials**


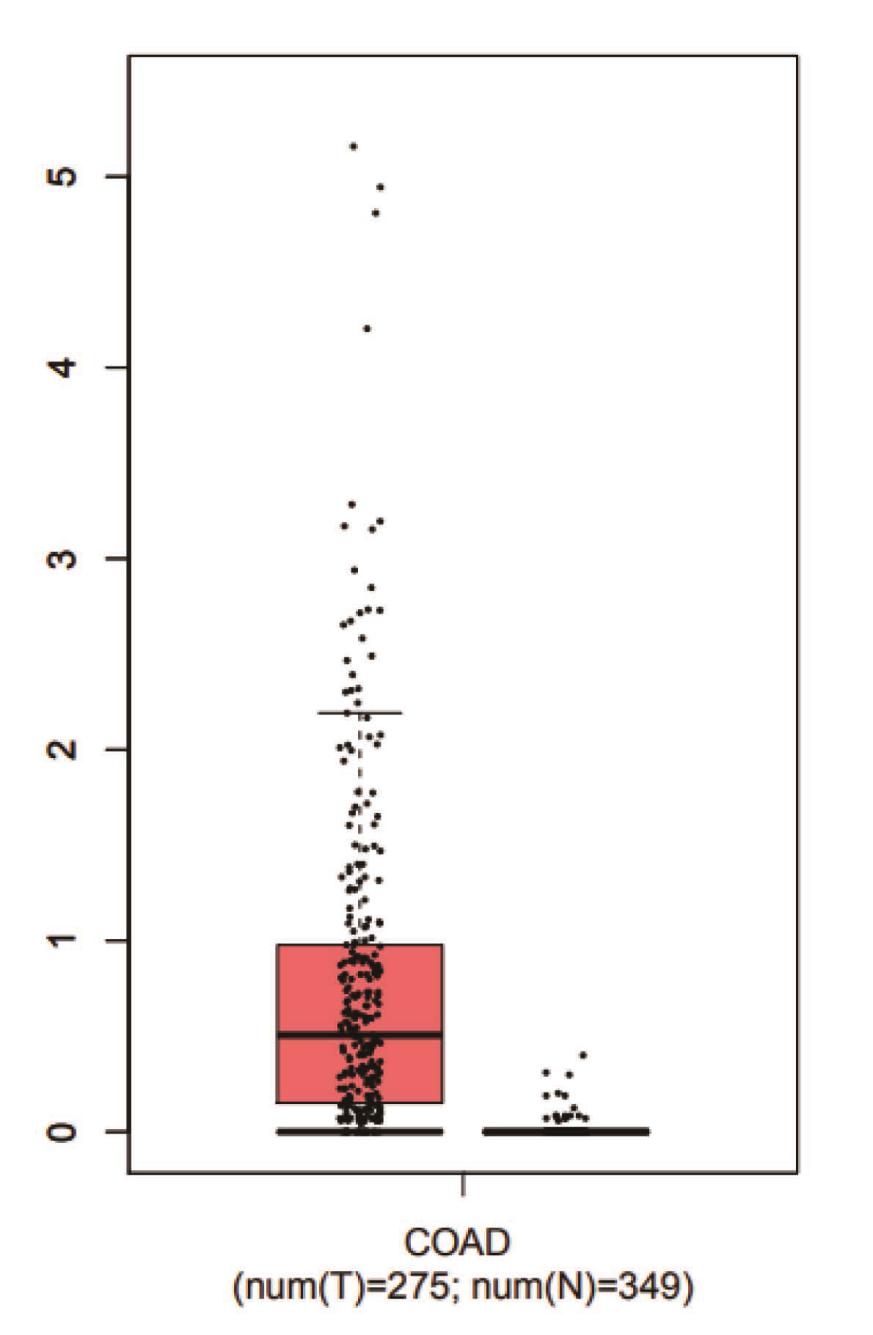


Supplementary Figure. 1 Expression of CST4 in TGAC database. The expression of CST4 in CRC tissue was significantly higher than that in normal tissues (P<0.05).
